# hnRNPM regulates influenza A virus replication through distinct mechanisms in human and avian cells: implications for cross-species transmission

**DOI:** 10.1128/jvi.00067-25

**Published:** 2025-05-28

**Authors:** Qin Zhang, Lei Zhang, Jinghua Li, Wenyu Zhang, Jianwei Wang, Tao Deng

**Affiliations:** 1 Laboratory of Pathogen Microbiology and Immunology, Institute of Microbiology, Chinese Academy of Sciences 85387, Beijing, China; 2 University of Chinese Academy of Sciences 74519, Beijing, China; 3 MOH Key Laboratory of Systems Biology of Pathogens, Institute of Pathogen Biology, Chinese Academy of Medical Sciences & Peking Union Medical College, Beijing, China; University of Kentucky College of Medicine, Lexington, Kentucky, USA

**Keywords:** influenza A virus, human hnRNPM, chicken hnRNPM, segment-specific transcription, M segment splicing

## Abstract

The eight-segmented RNA genome of influenza A virus (IAV) is transcribed and spliced into 10 major viral mRNAs in the nucleus of infected cells. Both transcription and splicing are facilitated by the host RNA polymerase II (Pol II) machinery via interactions between the viral ribonucleoprotein (vRNP) complex and various host factors. In this study, we demonstrate that IAV vRNPs recruit species-specific heterogeneous nuclear ribonucleoprotein M (hnRNPM) to support their replication in human and avian cells through distinct mechanisms. In A549 cells, human hnRNPM specifically facilitates the efficient transcription of HA, NA, M, and NS segments of WSN virus in a gene coding sequence-dependent manner. In contrast, in DF-1 cells, chicken hnRNPM restricts excessive splicing of M segment mRNA to ensure proper M2 protein production. Notably, human hnRNPM, with 34 additional amino acids compared with its chicken counterpart, fails to inhibit the M2 expression in DF-1 cells, whereas both human and chicken hnRNPM regulate WSN virus replication similarly in A549 cells. These findings highlight the host-specific roles of M2 levels in IAV replication and reveal how IAV co-opts host factors through virus genome sequence-dependent and host species-specific mechanisms, underscoring its high flexibility and adaptability during cross-species transmission.

**IMPORTANCE:**

The transcription and splicing of IAV genome in the nucleus of infected cells are precisely regulated to produce optimal amounts of viral proteins, ensuring efficient virus replication. In this study, we discovered that human hnRNPM regulates the IAV segment-specific differential transcription in a coding sequence-dependent manner in human cells. In contrast, chicken hnRNPM specifically inhibits M2 mRNA splicing to maintain proper M2 protein levels in avian cells. These species-specific regulatory mechanisms highlight the distinct replication strategies employed by IAV in human versus avian cells and underscore the complexity of cross-species transmission.

## INTRODUCTION

Influenza A viruses (IAVs) cause epidemics or pandemics in humans, resulting in significant public health risks and economic losses (1). IAV replication involves intricate interactions between the virus and numerous host proteins and RNAs, which are crucial for viral pathogenicity and transmissibility (2, 3). A comprehensive understanding of the mechanisms of influenza virus replication in host cells can significantly contribute to the prevention and treatment of influenza disease.

The genome of IAV consists of eight single-stranded negative-sense RNA segments, encoding at least 10 major viral proteins (PB2, PB1, PA, HA, NP, NA, M1, M2, NS1, and NS2) in which M2 and NS2 proteins are produced from the spliced transcripts of M and NS segments, albeit by different splicing mechanisms (4, 5). Among them, PB2, PB1, and PA form a heterotrimeric RNA-dependent RNA polymerase (RdRp) that binds to the viral RNA on the NP backbone to create a vRNP complex. Upon viral infection, HA and NA are responsible for the entry and release of virions, and HA and M2 promote the release of the viral genome into the cytosol. Then, eight vRNPs translocate to the nucleus and initiate the primary transcription of viral mRNA. Subsequently, newly synthesized polymerase subunits and NP enter the nucleus to initiate secondary mRNA transcription and vRNA replication (6). In the nucleus, the transcripts from the M and NS segment vRNAs are spliced to produce M2 and NS2, respectively. Notably, the mRNA transcripts from the M segment localize to nuclear speckles under the facilitation of the viral NS1 protein, for splicing and nuclear export, whereas the NS mRNA transcripts are more likely co-transcriptionally spliced in the nucleoplasm (5, 7). M1 and NS2 mediate the nuclear export of vRNPs, which are subsequently assembled into new virus particles (6). NS1 has multiple functions that are critical for viral replication (8). Each of these viral proteins is indispensable for the life cycle of the influenza virus, and their expression is finely and precisely regulated in host cells.

The expression of the IAV genome exhibits segmental differences. HA, NP, NA, M, and NS segments are transcribed into mRNAs at significantly higher levels than the PB2, PB1, and PA segments (9–11). This differential transcription results in the production of viral proteins at various levels, which are associated with their respective functions during IAV replication (10, 12). Transcription initiation and the subsequent fate of viral mRNA depend on the regulation of host factors (3, 13, 14). Several studies have implicated host factors and viral proteins in the differential post-transcriptional regulation of influenza viruses. Examples include the specific regulation of M mRNA splicing by hnRNPK (15), the differential regulation of viral mRNA nuclear export by NXF1/TAP (16), the specific regulation of HA, M, and NS translation by ZFP36L1 (17), and the involvement of the viral protein NS1 in the differential expression of IAV proteins (18, 19). However, the factors that regulate the differential transcription of the IAV genome remain unidentified.

Due to the limited availability of viral RNA-binding proteins (RBPs), RNA viruses depend on numerous cellular RBPs throughout their life cycle to facilitate replication. Heterogeneous nuclear ribonucleoprotein M (hnRNPM), an essential RNA-binding protein, is a component of the spliceosome complex (20), may interact with RNA polymerase II (Pol II) (15), and localizes to nuclear speckles enriched with splicing factors (21). Previous studies have shown that hnRNPM is involved in the replication of several viruses, including Sindbis virus (SINV), vesicular stomatitis virus (VSV), Newcastle disease virus (NDV), and encephalomyocarditis virus (EMCV) (22). Importantly, proteomic analyses from different studies have suggested a potential interaction between hnRNPM and the vRNP components of IAV (23–25). Knockdown of hnRNPM has been reported to inhibit the replication of A/VN/1203/04 (H5N1) virus (26). However, the specific role of hnRNPM in the IAV life cycle remains unclear. Notably, hnRNPM features three conserved RNA recognition motifs (RRM) and has species-specific auxiliary domains that vary between human and chicken orthologs. Differences between human and chicken orthologs of specific proteins, including acidic nuclear phosphoprotein 32 (ANP32A), myxovirus resistance protein 1 (MX1), and butyrophilin subfamily 3 member A3 (BTN3A3), are well-documented as barriers to cross-species transmission of influenza viruses (27–29). Given the ongoing spread of cow H5N1 and the increasing risk of transmission to humans, understanding the impact of species-specific host factors on viral replication has become particularly important. Whether species-specific hnRNPM variations contribute to the cross-species barriers for IAV remains unknown.

In this study, we found that human hnRNPM (hu-hnRNPM) differentially regulates IAV genome transcription in human cells, specifically modulating the transcription of HA, NA, M, and NS segments. By contrast, in DF-1 cells, chicken hnRNPM (ch-hnRNPM) inhibits the splicing of M mRNA, resulting in lower levels of M2 mRNA and protein, a function not observed with hu-hnRNPM. This species-specific regulation by hnRNPM leads to significant differences in M2 protein levels between human and avian cells. These species-specific differences in hnRNPM regulation not only emphasize the importance of host-specific M2 levels in IAV replication but also underscore a critical barrier to IAV cross-species transmission between humans and birds.

## RESULTS

### hnRNPM interacts with IAV vRNP as identified by affinity purification

**Fig 1:**
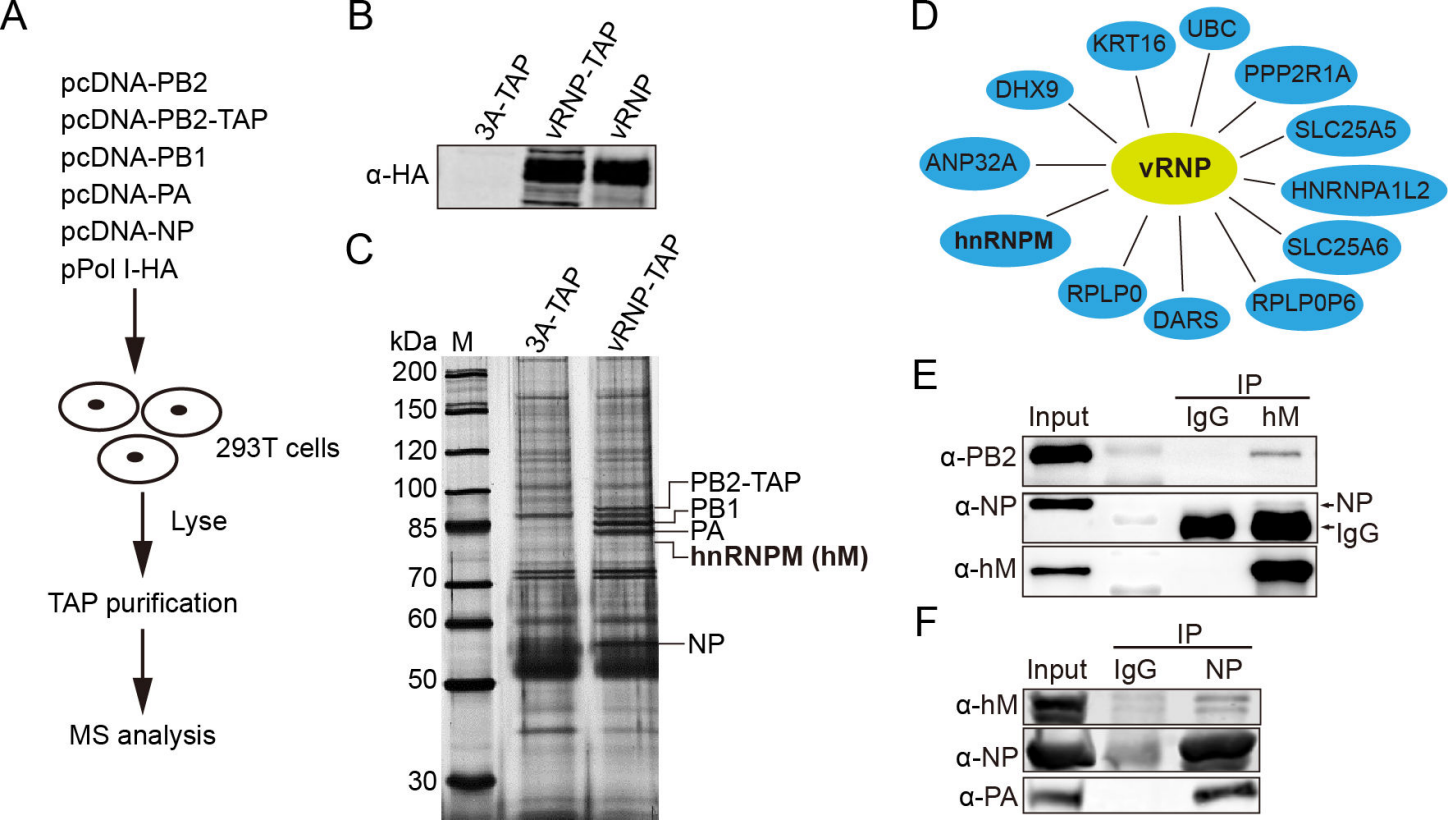
hnRNPM interacts with IAV vRNP as identified by affinity purification. (**A**) Schematic of the AP-MS screen to investigate influenza virus RNP interactions with host factors. (**B**) Polymerase activity is not affected by the vRNP with PB2-TAP. Recombinant HA-containing viral vRNP was generated using either PB2 or PB2-TAP. HA protein was assessed using western blot analysis using anti-HA antibodies. (**C**) Analysis of the purification of the recombinant TAP-tagged RNP complex. Cells were harvested and lysed 24 h post-transfection, followed by purification using IgG-Sepharose. The purified proteins were separated by 8% SDS-PAGE and visualized by silver staining. The positions of PB2-TAP, PB1, PA, NP, and hnRNPM are indicated. (**D**) Cellular proteins identified by TAP purification of the vRNP complex. The purified proteins were subjected to LC-MS/MS analysis, resulting in the identification of 12 host proteins with high confidence and at least two unique peptide identifications per protein. (**E, F**) Interaction between hnRNPM and viral RNP confirmed by co-immunoprecipitation. A549 cells were infected with WSN virus at an MOI of 1 for 12 h. Cell lysates were immunoprecipitated with either an anti-hnRNPM antibody (**E**) or an anti-NP antibody (**F**), followed by Western blot analysis.

Influenza virus replication in host cells relies on numerous host factors. To better understand how the virus exploits these host proteins, we employed affinity purification coupled with mass spectrometry (AP-MS) to identify host proteins that interact with influenza vRNP complexes (Fig. 1A). 293T cells were transfected with plasmids encoding polymerase subunits (PB1, PA, and either PB2 or PB2-TAP), NP, and a plasmid expressing HA vRNA (pPOLI-HA) to produce HA vRNP of influenza A/WSN/33 (WSN, H1N1) virus. The polymerase, including PB2-TAP, was fully functional for HA expression (Fig. 1B). After 24 h post-transfection, proteins from cells were subjected to affinity purification using a TAP antibody to isolate vRNP-bound proteins. The purified proteins were separated by 8% SDS-PAGE, visualized by silver staining (Fig. 1C), and identified by mass spectrometry.

Our AP-MS analysis identified 12 host proteins that interact with vRNP with high confidence (Fig. 1D). These include ANP32A, a protein reported to play a critical role during viral RNA replication and restrict host adaptation (30), as well as RNA-binding proteins such as hnRNPM, hnRNPA1L2, DARS, and DHX9. A comparison with previous proteomic studies (23–25) further revealed potential interactions between hnRNPM and vRNP. Based on this, we focused on hnRNPM and sought to validate its interaction with viral RNPs through co-immunoprecipitation (co-IP) experiments during IAV infection. Using a specific antibody against hnRNPM, we successfully co-immunoprecipitated vRNP complexes, including PB2 and NP (Fig. 1E). Similarly, hnRNPM was co-precipitated with an NP-specific antibody, with PA serving as a positive control for vRNP co-precipitation (Fig. 1F). These results confirm that hnRNPM interacts with influenza vRNP, suggesting that hnRNPM may play a critical role in IAV infection.

### Human hnRNPM positively regulates IAV replication in human cells

**Fig 2:**
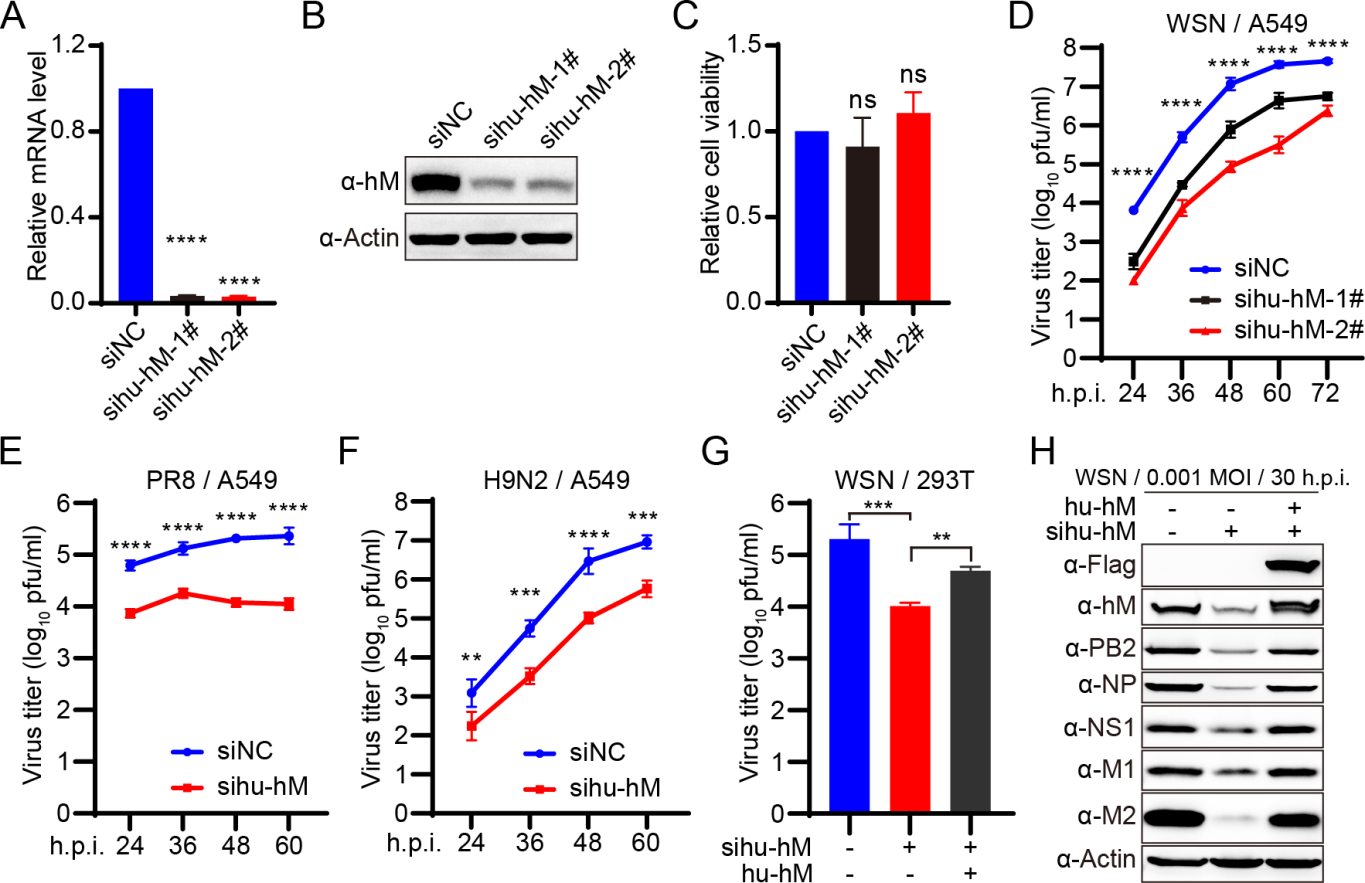
Human hnRNPM positively regulates IAV replication in human cells. (**A, B**) SiRNA knockdown of hnRNPM in A549 cells. A549 cells were transfected with two individual siRNAs targeted to hu-hnRNPM (sihu-hM-1# and sihu-hM-2#) or a scrambled siRNA as a negative control (siNC) for 48 h. The mRNA (**A**) and protein (**B**) levels of hnRNPM were quantified by RT-qPCR and western blotting, respectively. ****P < 0.0001. (**C**) Cell viability of sihu-hnRNPM-treated A549 cells was assessed using a Cell Counting Kit-8 (CCK-8) assay. ns, not significant. (D–F) Growth curves of WSN, PR8, and H9N2 viruses in A549 cells. A549 cells were treated with the indicated siRNAs for 36 h and then infected with WSN (MOI = 0.001) (**D**), A/Puerto Rico/8/34 (PR8, H1N1) (MOI = 0.1) (**E**), or H9N2 virus (MOI = 0.01) (**F**). Supernatants were collected at the indicated time points and subjected to plaque assays on MDCK cells. **P < 0.01, ***P < 0.001, ****P < 0.0001. (**G, H**) Overexpression of hu-hnRNPM restores the replication of WSN virus in sihu-hnRNPM-treated 293T cells. Sihu-hnRNPM- or siNC-treated 293T cells were transfected with the plasmids encoding either an empty vector or hu-hnRNPM (hu-hM). At 24 h post-transfection, the cells were infected with WSN virus at an MOI of 0.001 for 30 h post-infection. Virus titers in the supernatants were determined by performing plaque assays (**G**), and whole-cell lysates were analyzed by Western blotting (**H**). Data are representative of at least three independent experiments. Means ± SD are shown in (**A, **C–G) (*n* = 3).

To investigate the role of hnRNPM in IAV replication, we performed siRNA-knockdown experiments in A549 cells, followed by infection with the WSN virus. SiRNA targeting human hnRNPM (hu-hnRNPM) efficiently silenced its expression, as confirmed by quantitative reverse transcription PCR (RT-qPCR) (Fig. 2A) and western blotting (Fig. 2B), without affecting cell viability (Fig. 2C). Knockdown of hu-hnRNPM led to more than a 10-fold reduction in viral titers from 24 to 72 h post-infection (Fig. 2D), indicating its critical role in WSN virus replication. Additionally, the role of hu-hnRNPM in IAV replication was confirmed with A/Puerto Rico/8/34 (PR8, H1N1) and A/Quail/Hong Kong/G1/1997 (H9N2) viruses. The growth titers of both strains were similarly reduced by more than 10-fold in hu-hnRNPM knockdown cells from 24 to 60 h post-infection (Fig. 2E and F), further supporting a role of hu-hnRNPM in the efficient replication of multiple IAV subtypes in human cells.

Next, we complemented hu-hnRNPM expression in sihu-hnRNPM-treated 293T cells by transfecting a plasmid encoding hu-hnRNPM with synonymous mutations in the siRNA-targeted codon. Re-expression of hu-hnRNPM restored WSN virus titers in supernatants and viral protein levels in cells (Fig. 2G and H), demonstrating that the inhibitory effect of hu-hnRNPM knockdown on influenza virus replication is directly attributable to the loss of hu-hnRNPM.

### Human hnRNPM regulates IAV replication independently of STAT1-mediated innate immune pathways

**Fig 3:**
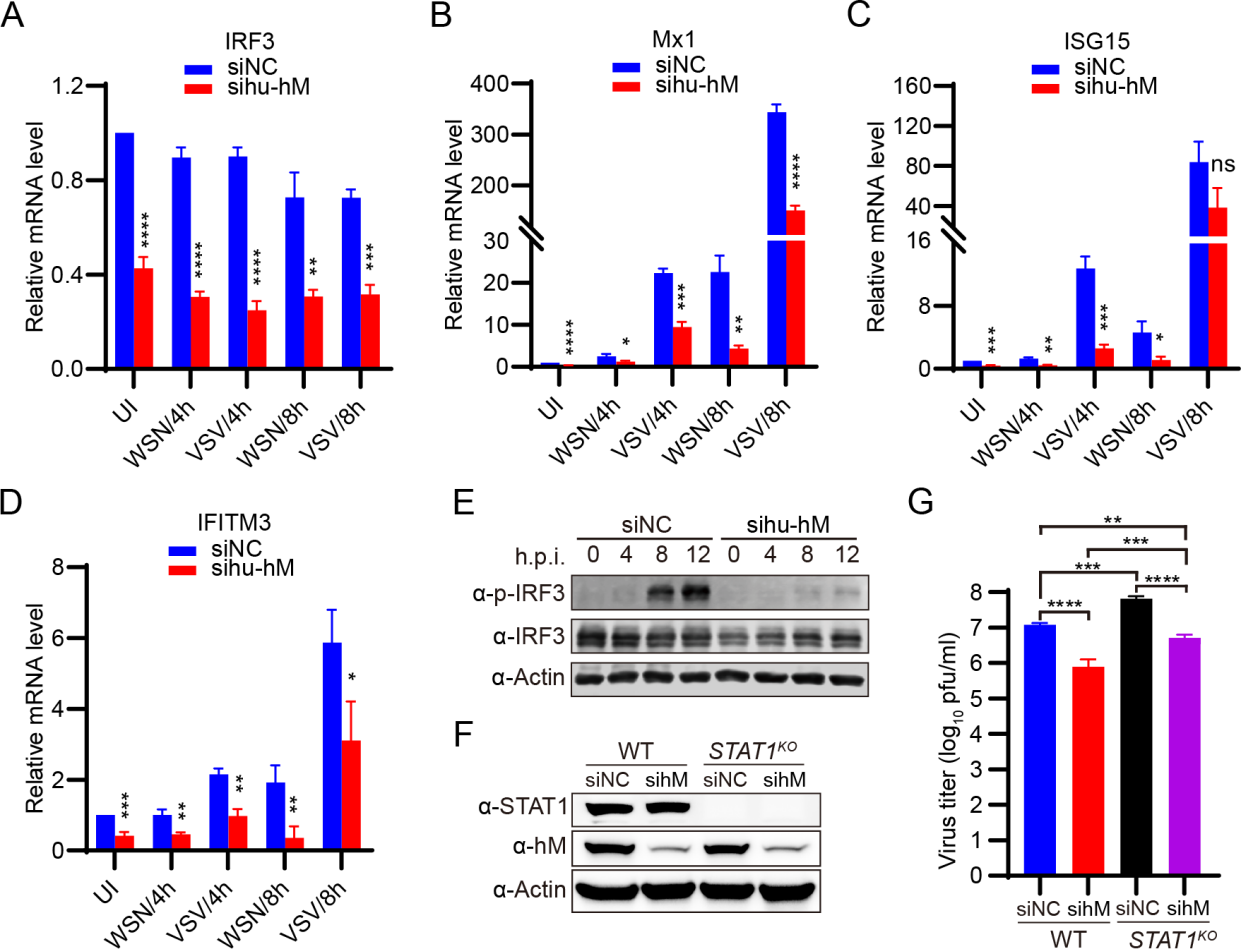
Human hnRNPM regulates IAV replication independently of STAT1-mediated innate immune pathways**.** (A–D) Effect of hu-hnRNPM knockdown on IRF3 (**A**) and ISGs (B–D) transcripts in A549 cells. A549 cells were transfected with the indicated siRNAs for 36 h, then uninfected (UI) or infected with WSN virus (MOI = 1) or VSV virus (MOI = 3). Cellular mRNAs were analyzed using qPCR at the indicated time points. *P < 0.05, **P < 0.01, ***P < 0.001, ****P < 0.0001; ns, not significant. (**E**) Hu-hnRNPM knockdown affects IRF3 and its phosphorylated protein levels. A549 cells were treated with the indicated siRNA, infected with WSN virus, and subsequently subjected to western blot analysis using specific antibodies. (**F**) Analysis of STAT1-knockout and hnRNPM knockdown in A549 cells using immunoblotting. (**G**) Knockdown of hnRNPM reduces WSN virus replication in STAT1-knockout A549 cells. A549 cells and STAT1-knockout A549 cells, with or without hu-hnRNPM knockdown, were infected with WSN virus (MOI = 0.001) for 48 h post-infection. Virus titers in the supernatants were quantified using the plaque assay. ***P < 0.001, ****P < 0.0001. Data are representative of at least three independent experiments. Means ± SD are shown in (A–D, G) (*n* = 3).

Previous studies have shown that hnRNPM may regulate innate immune responses, although conflicting evidence exists. hnRNPM depletion enhances innate immune gene expression in HeLa, 293T, and mouse macrophage cell lines (31, 32), whereas other reports demonstrate that hnRNPM knockdown suppresses innate immune responses in 293T cells (33, 34). This apparent discrepancy may arise from cell type-specific regulatory mechanisms. Since A549 cells are a more physiologically relevant model for influenza virus infection, we investigated whether hnRNPM’s regulation of IAV replication is mechanistically linked to innate immune gene expression in these cells. We found that hu-hnRNPM knockdown reduced the transcription of IRF3 and interferon-stimulated genes (ISGs), including Mx1, ISG15, and IFITM3, both in the absence and presence of WSN or VSV virus infection in A549 cells (Fig. 3A through D). Moreover, hu-hnRNPM knockdown decreased IRF3 protein levels at 0, 4, and 8 h post-infection and reduced the phosphorylation of IRF3 induced by IAV infection at 8 and 12 h (Fig. 3E). These results suggest that hu-hnRNPM regulates the expression of innate immune genes, which is consistent with the recent findings of Alexander Kirchhoff and colleagues (33). However, the inhibition of IAV replication following hu-hnRNPM knockdown may involve mechanisms beyond innate immune regulation.

To further validate these findings, we assessed the impact of hnRNPM on IAV replication in cells with defective innate immune pathways. Given that STAT1 activates the expression of numerous ISGs in response to upstream immune signals (35), we depleted hu-hnRNPM in *STAT1*-knockout A549 cells (Fig. 3F) and infected them with WSN virus. As expected, hu-hnRNPM knockdown in *STAT1*-knockout A549 cells resulted in significantly reduced WSN virus titers compared with both *STAT1*-knockout cells and wild-type control cells (Fig. 3G). These findings suggest that IAV replication is regulated by hu-hnRNPM independently of the STAT1-dependent innate immune responses.

### Human hnRNPM differentially regulates the transcription efficiency of the IAV genome in a segment-specific manner

**Fig 4:**
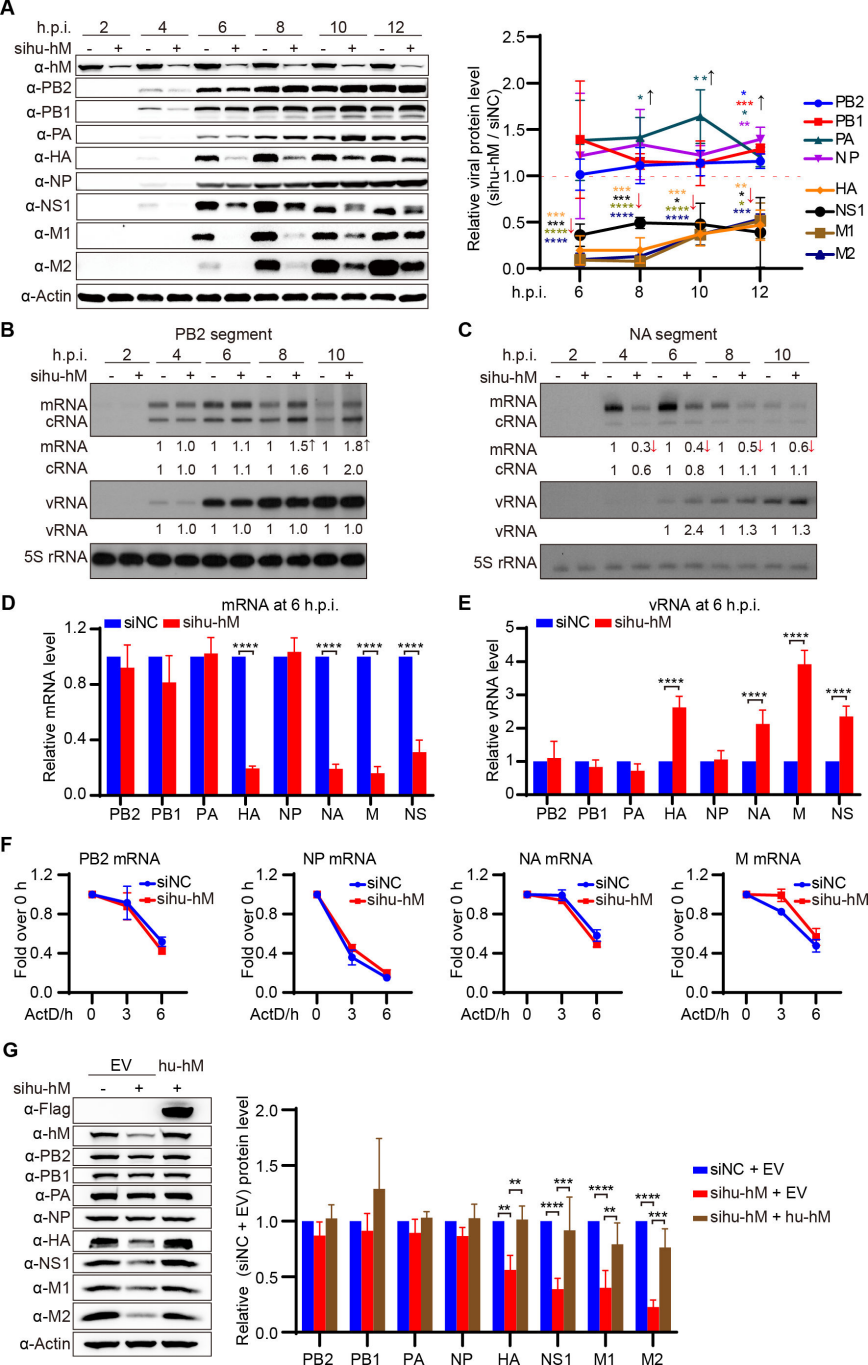
Human hnRNPM differentially regulates the transcription efficiency of the IAV genome in a segment-specific manner**.** (**A-C**) Hu-hnRNPM knockdown results in the differential expression of IAV genes in A549 cells. A549 cells were transfected with the indicated siRNAs for 36 h and infected with the WSN virus at an MOI of 10. Cells were harvested at specific time points. Proteins were analyzed by western blotting (**A**). Quantification of viral proteins is shown in the right panel. RNAs were analyzed using primer extension analysis to detect the vRNA, mRNA, and cRNA of PB2 (**B**) and NA (**C**) segments. *P < 0.05, **P < 0.01, ***P < 0.001, ****P < 0.0001. (**D, E**) Quantification of IAV eight-gene mRNA (**E**) and vRNA (**F**) by qPCR analysis at 6 h post-infection. ****P  <  0.0001. (**F**) Hu-hnRNPM does not regulate IAV mRNA degradation. 293T cells were transfected with the indicated siRNA and subsequently transfected with expression plasmids (pcDNA) encoding PB2, NP, NA, and M. After 24 h, total RNAs were harvested at 0, 3, and 6 h post-actinomycin D treatment, and the mRNA levels of PB2, NP, NA, and M were assessed by qPCR analysis. ns, not significant. (**G**) Overexpression of hu-hnRNPM restores the differential expression of the WSN virus protein in sihu-hnRNPM-treated A549 cells. Sihu-hnRNPM- or siNC-treated A549 cells were transfected with the plasmids encoding either an empty vector (EV) or hu-hnRNPM (hu-hM). At 24 h post-transfection, the cells were infected with WSN virus at an MOI of 10. Cells were harvested at 7 h post-infection. Proteins were analyzed by western blotting. Quantification of viral proteins is shown in the right panel. **P < 0.01, ***P < 0.001, ****P < 0.0001. Data are representative of at least three independent experiments. Means ± SD are shown in (**A, D-G**) (*n* = 3).

To define the specific role of hu-hnRNPM in IAV replication, we investigated its effect on IAV genome expression in a single round of WSN virus infection. Viral proteins were detected by western blotting using specific antibodies, except for WSN NA and NS2, which lacked suitable antibodies (Fig. 4A). We found that hu-hnRNPM knockdown significantly reduced the expression of HA, M1, NS1, and M2 proteins at all time points. In contrast, PB2, PB1, PA, and NP proteins exhibited slight increases at 10 and 12 h post-infection, suggesting that hu-hnRNPM plays a critical role in differentially regulating the expression of the IAV genome.

Next, we analyzed viral RNA levels in the single-round WSN virus infection, focusing on the mRNA and vRNA of the PB2 and NA segments using primer extension analysis (Fig. 4B and C). We found a positive correlation between viral proteins and mRNAs, but not with vRNAs. Specifically, hu-hnRNPM knockdown slightly increased PB2 mRNA levels at 8 and 10 h post-infection, while significantly decreasing NA mRNA levels at all time points. Hu-hnRNPM knockdown did not alter PB2 vRNA levels but modestly increased NA vRNA levels. To extend our analysis, we employed RT-qPCR to measure the mRNA and vRNA levels of all eight IAV segments at 6 h post-infection. Knockdown of hu-hnRNPM significantly reduced the mRNA levels of HA, NA, M, and NS, without effect on PB2, PB1, PA, and NP segments (Fig. 4D). Although hu-hnRNPM depletion did not alter vRNA levels for PB2, PB1, PA, and NP segments, it resulted in elevated vRNA levels of HA, NA, M, and NS segments (Fig. 4E). These results suggest that hu-hnRNPM knockdown leads to segment-specific differential expression of viral RNA, with a negative correlation between changes in mRNA and vRNA levels for HA, NA, M, and NS genes. Additionally, we observed that hu-hnRNPM depletion did not affect the degradation rate of viral mRNAs (Fig. 4F). Collectively, these results indicate that hu-hnRNPM knockdown differentially regulates the transcription of influenza virus genes.

To further confirm the role of hu-hnRNPM in the differential expression of IAV, we complemented hu-hnRNPM expression in sihu-hnRNPM-treated A549 cells and assessed its impact on viral protein expression. We found that restoring hu-hnRNPM expression in hu-hnRNPM-knockdown cells rescued the expression of HA, NS1, M1, and M2 (Fig. 4G), demonstrating that the reduction in the expression of these genes in sihu-hnRNPM-treated cells is directly attributable to hu-hnRNPM depletion. Together, our results confirm that hu-hnRNPM differentially regulates the transcription efficiency of the IAV genome in a segment-specific manner, leading to variable levels of viral proteins.

### Human hnRNPM regulates the segment-specific transcription efficiency in a coding sequence-specific manner

**Fig 5:**
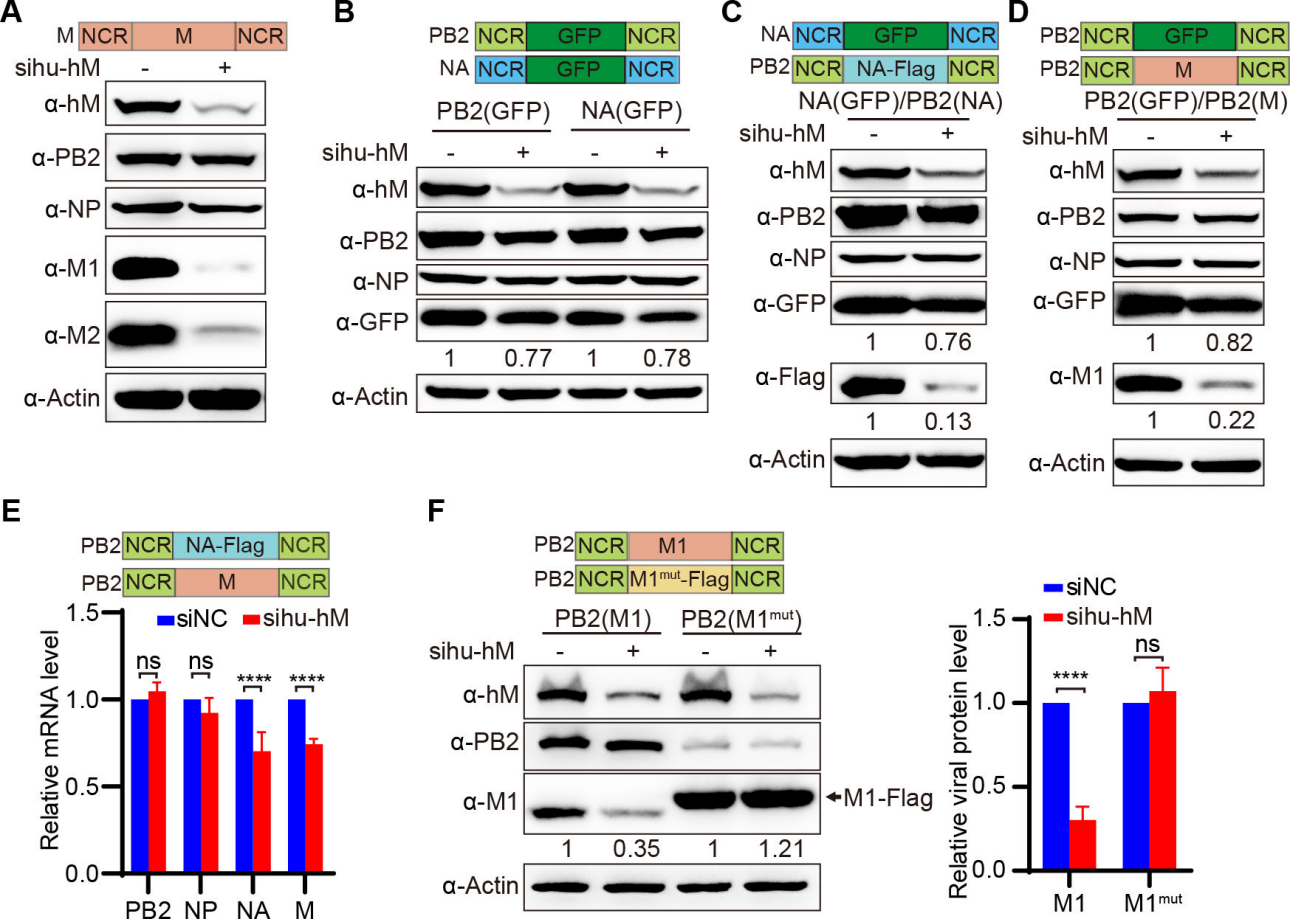
Human hnRNPM regulates the segment-specific transcription efficiency in a coding sequence-specific manner. (**A**) Hu-hnRNPM specifically inhibits M1 and M2 expression in an RNP reconstitution system. 293T cells were treated with the indicated siRNAs, then transfected with bidirectional expression plasmids pHW2000 encoding PB2, PB1, PA, and NP, along with Pol I-driven RNA expression plasmids encoding M vRNA. Cellular proteins were analyzed by immunoblotting. (**B**) Hu-hnRNPM regulates viral gene expression independently of the viral gene’s NCR. 293T cells were transfected with the indicated siRNAs and RNP reconstitution plasmids. Pol I-driven RNA expression plasmids encode chimeric PB2(GFP) or NA(GFP) RNAs. Proteins were analyzed by immunoblotting. (C–F) Hu-hnRNPM regulates viral gene expression based on the ORF sequence. siRNA-treated 293T cells were transfected with RNP reconstitution plasmids and Pol I-driven recombinant plasmids. These Pol I-driven plasmids retained the NCRs of PB2 or NA and replaced their ORFs with GFP, NA, M, or M1. Specifically, the Pol I-driven plasmids encoded the following chimeric RNAs: NA(GFP) and PB2(NA-Flag) in (**C**), PB2(GFP) and PB2(M) in (**D**), PB2(NA-Flag) and PB2(M) in (**E**), PB2(M1) and PB2(M1^mut^-Flag) in (**F**). Cellular proteins were analyzed by immunoblotting in (C, D, and F). mRNAs were identified by qPCR analysis in (**E**). Each protein band was quantified by ImageJ. ****P < 0.0001; ns, not significant. Data are representative of at least three independent experiments. Means ± SD are shown in (**E, F**) (*n* = 3).

The genetic structure of each influenza viral RNA segment consists of specific open reading frames (ORFs) flanked by non-coding regions (NCRs) at both ends. These NCRs contain a conserved promoter region and segment-specific sequences and are essential for regulating viral transcription, replication, and protein expression (36–41). To investigate whether the regulation by hu-hnRNPM relies on specific regions of the IAV gene sequences, we examined the effects of hnRNPM in RNP reconstitution systems with various chimeric templates. Our results show that knocking down hu-hnRNPM reduces the levels of M1 and M2 proteins but does not affect the expression of PB2 and NP proteins, consistent with observations from viral infection (Fig. 5A and 4A). This approach allows us to effectively analyze the impact of different viral gene regions on hu-hnRNPM regulation.

We first constructed chimeric vRNAs, PB2(GFP) and NA(GFP), which preserve their NCRs while replacing the ORF with GFP (Fig. 5B). Knockdown of hu-hnRNPM led to a modest reduction in GFP expression from both PB2(GFP) and NA(GFP) vRNAs, with similar levels of reduction, suggesting that hu-hnRNPM regulation of viral gene expression is independent of NCR sequences. Consequently, we hypothesized that the ORF sequences are critical for hu-hnRNPM-mediated regulation of viral gene expression. To test this, we substituted the ORF of PB2 with those from the NA or M segments to generate chimeric vRNAs, namely PB2(NA-Flag) and PB2(M). Hu-hnRNPM knockdown significantly reduced NA-Flag protein expression from PB2(NA-Flag) compared with GFP expression from NA(GFP) (Fig. 5C). Similarly, hu-hnRNPM depletion markedly reduced M1 protein expression from PB2(M) relative to GFP expression from PB2(GFP) (Fig. 5D). Consistent with protein expression, hu-hnRNPM knockdown significantly reduced the mRNA levels of NA and M transcribed from PB2(NA-Flag) and PB2(M) (Fig. 5E). These results support our hypothesis that hu-hnRNPM regulates viral gene expression based on ORF sequences rather than NCR sequences. To further confirm this, we constructed a chimeric vRNA, PB2(M1^mut^-Flag), containing synonymous mutations in the M1 codons. Specifically, out of the total 759 nucleotides encoding the M1 protein, 178 were altered, with these mutations evenly distributed throughout the entire coding region. Notably, hu-hnRNPM knockdown no longer inhibited M1-Flag expression from PB2(M1^mut^-Flag), in contrast to the significant decrease in M1 expression from PB2(M1) (Fig. 5F). Taken together, these results definitively demonstrate that hu-hnRNPM regulates viral gene expression based on ORF sequences.

### Chicken hnRNPM inhibits the splicing of IAV M2 mRNA to support IAV replication in DF-1 cells

**Fig 6:**
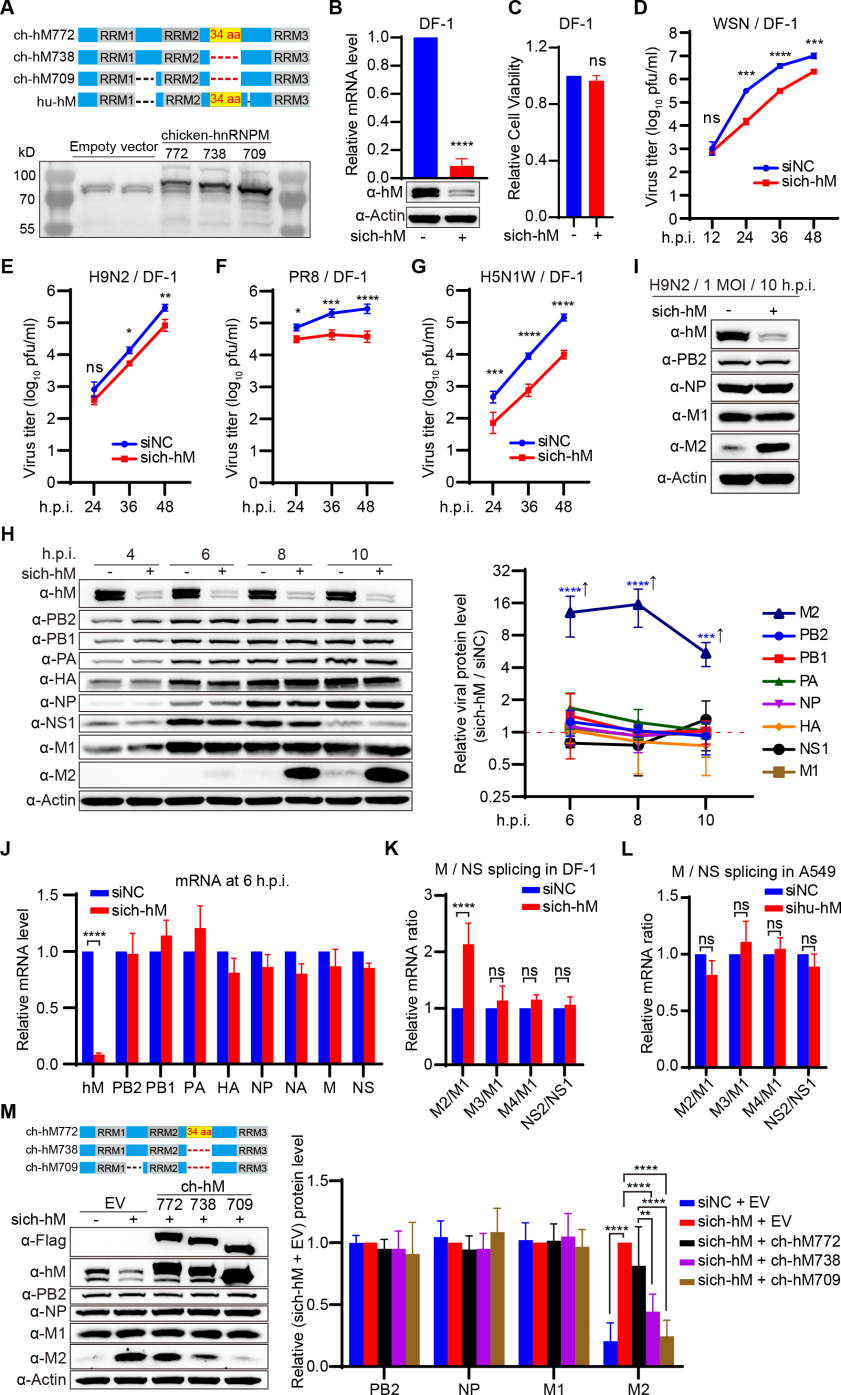
Chicken hnRNPM inhibits the splicing of IAV M2 mRNA to support IAV replication in DF-1 cells. (**A**) Identify the predominantly expressed chicken hnRNPM isoforms in DF-1 cells. The protein sequences of human hnRNPM and three predicted isoforms of chicken hnRNPM (ch-hM772, ch-hM738, and ch-hM709, named according to their amino acid lengths) were aligned to identify conserved domains and species-specific domains among the orthologs. Based on the alignment results, plasmids expressing the three predicted isoforms of chicken hnRNPM were transfected into DF-1 cells, and the predominantly expressed isoform in DF-1 cells was determined using hnRNPM-specific antibodies. (**B**) SiRNA knockdown of ch-hnRNPM (ch-hM) in DF-1 cells. DF-1 cells were transfected with control siRNA (siNC) or ch-hnRNPM-specific siRNA (sich-hM) for 48 h. The mRNA and protein levels of ch-hnRNPM were quantified by RT-qPCR and western blotting. ****P < 0.0001. (**C**) Cell viability of sich-hM-treated DF-1 cells was assessed using a CCK-8 assay. ns, not significant. (D–G) Growth curves of WSN, H9N2, PR8, and H5N1W viruses in DF-1 cells. DF-1 cells treated with the indicated siRNAs were infected with 0.001 MOI of WSN (**D**), 0.001 MOI of H9N2 (**E**), 0.1 MOI of PR8 (**F**), and 0.001 MOI of H5N1W (**G**). Supernatants were collected at the indicated time points post-infection, and virus titers were determined by plaque assay using MDCK cells. *P < 0.05, **P < 0.01, ***P < 0.001, ****P < 0.0001; ns, not significant. (**H, I**) Knockdown of ch-hnRNPM specifically increases M2 expression in DF-1 cells. DF-1 cells were transfected with the indicated siRNAs and infected with WSN (**H**) or H9N2 (**I**) viruses at an MOI of 10. Cells were harvested at specific time points, and proteins were analyzed by Western blotting. Quantification of WSN viral proteins is shown in the right panel. ****P  <  0.0001; ns, not significant. (**J**) Detection of IAV eight-gene mRNA by qPCR analysis at 6 h post-infection. mRNAs from (**H**) cells were quantified using RT-qPCR. ****P  <  0.0001; ns, not significant. (**K, L**) The splicing ratios of M and NS mRNA in DF-1 (**K**) and A549 cells (**L**). DF-1 and A549 cells were treated with control or targeting host-specific hnRNPM siRNAs, followed by infection with WSN virus at an MOI of 10. Total RNA was harvested at 6 h post-infection, and the splicing ratios of the M and NS segments were determined using RT-qPCR. ****P  <  0.0001; ns, not significant. (**M**) Overexpression of ch-hnRNPM re-inhibits the expression of M2 protein in sich-hnRNPM-treated DF-1 cells. DF-1 cells treated with either sich-hnRNPM or siNC were transfected with the indicated plasmids. At 24 h post-transfection, the cells were infected with WSN virus at an MOI of 10. Cells were harvested at 8 h post-infection, and viral proteins were analyzed by Western blotting. Quantification of viral proteins is shown in the right panel. **P < 0.01, ***P < 0.001, ****P < 0.0001. Data are representative of at least three independent experiments. Means ± SD are shown in (B–H, J–M) (*n* ≥ 3).

Transmission of IAV between humans and birds can result in epidemics or even pandemics. Species-specific proteins, such as ANP32A, MX1, and BTN3A3, are known to play a critical role in determining the cross-species transmission of IAV (27–29). The protein sequence alignment of human hnRNPM and the three predicted isoforms of chicken hnRNPM (ch-hM772, ch-hM738, and ch-hM709, named according to their amino acid lengths) reveals three conserved RNA recognition motifs (RRM1, RRM2, and RRM3), along with species-specific deletion sequences (Fig. 6A). In DF-1 cells, the predominantly expressed isoforms are the ch-hM738 and ch-hM709 variants, which lack a 34-amino acid (34 aa) sequence present in the predicted ch-hM772 isoform and human hnRNPM (Fig. 6A). It is of interest to investigate whether these specific deletions, analogous to those observed in ANP32A or MX1, affect influenza virus replication. Thus, we investigated the impact of depleting chicken hnRNPM (ch-hnRNPM) on IAV replication in DF-1 cells. Silencing ch-hnRNPM using siRNA effectively reduced both the mRNA and protein levels of hnRNPM in DF-1 cells, without affecting cell viability (Fig. 6B and C). Notably, knockdown of ch-hnRNPM resulted in a significant reduction in the titers of WSN, H9N2, PR8, and H5N1W (the HA and NA of the H5N1W recombinant virus are derived from WSN) viruses at 24 to 48 h post-infection (Fig. 6D through G), suggesting that ch-hnRNPM also plays a critical role in IAV replication.

To investigate whether the role of ch-hnRNPM in IAV replication is similar to that of hu-hnRNPM in human cells, we examined the effect of ch-hnRNPM depletion on viral gene expression in a single round of infection in DF-1 cells. Surprisingly, knockdown of ch-hnRNPM did not affect the differential expression of viral proteins. Instead, it significantly increased WSN viral M2 protein expression compared to control cells (Fig. 6H), a result that differs from observations in human cells. Similarly, ch-hnRNPM knockdown also elevated H9N2 viral M2 protein expression (Fig. 6I). To further investigate, we assessed viral RNA levels at 6 h post-infection and observed that depletion of ch-hnRNPM did not affect the mRNA levels of the eight viral segments (Fig. 6J). These results led us to hypothesize that ch-hnRNPM might influence the splicing of IAV mRNA. Indeed, knockdown of ch-hnRNPM significantly increased the M2/M1 mRNA ratio but had no effect on the M3/M1, M4/M1, or NS2/NS1 mRNA ratios (Fig. 6K). In contrast, knockdown of hu-hnRNPM in A549 cells had no impact on these splicing ratios (Fig. 6L). These results suggest that depletion of ch-hnRNPM enhances the splicing of M2 mRNA, resulting in increased M2 protein levels in DF-1 cells.

To further confirm the role of ch-hnRNPM, we reintroduced its expression in ch-hnRNPM-depleted DF-1 cells by transfecting plasmids encoding three potential isoforms of ch-hnRNPM (ch-hM772, ch-hM738, and ch-hM709). Among these isoforms, the supplementation of ch-hM738 and ch-hM709, predominantly expressed in DF-1 cells, significantly reduced M2 protein levels compared to the ch-hM772 isoform (Fig. 6M). These results indicate that ch-hnRNPM primarily regulates M2 mRNA splicing in DF-1 cells, suggesting a distinct mechanism of action compared with its human counterpart in human cells.

### Species-specific regulation of M2 expression by hnRNPM influences IAV replication in different host cells

**Fig 7:**
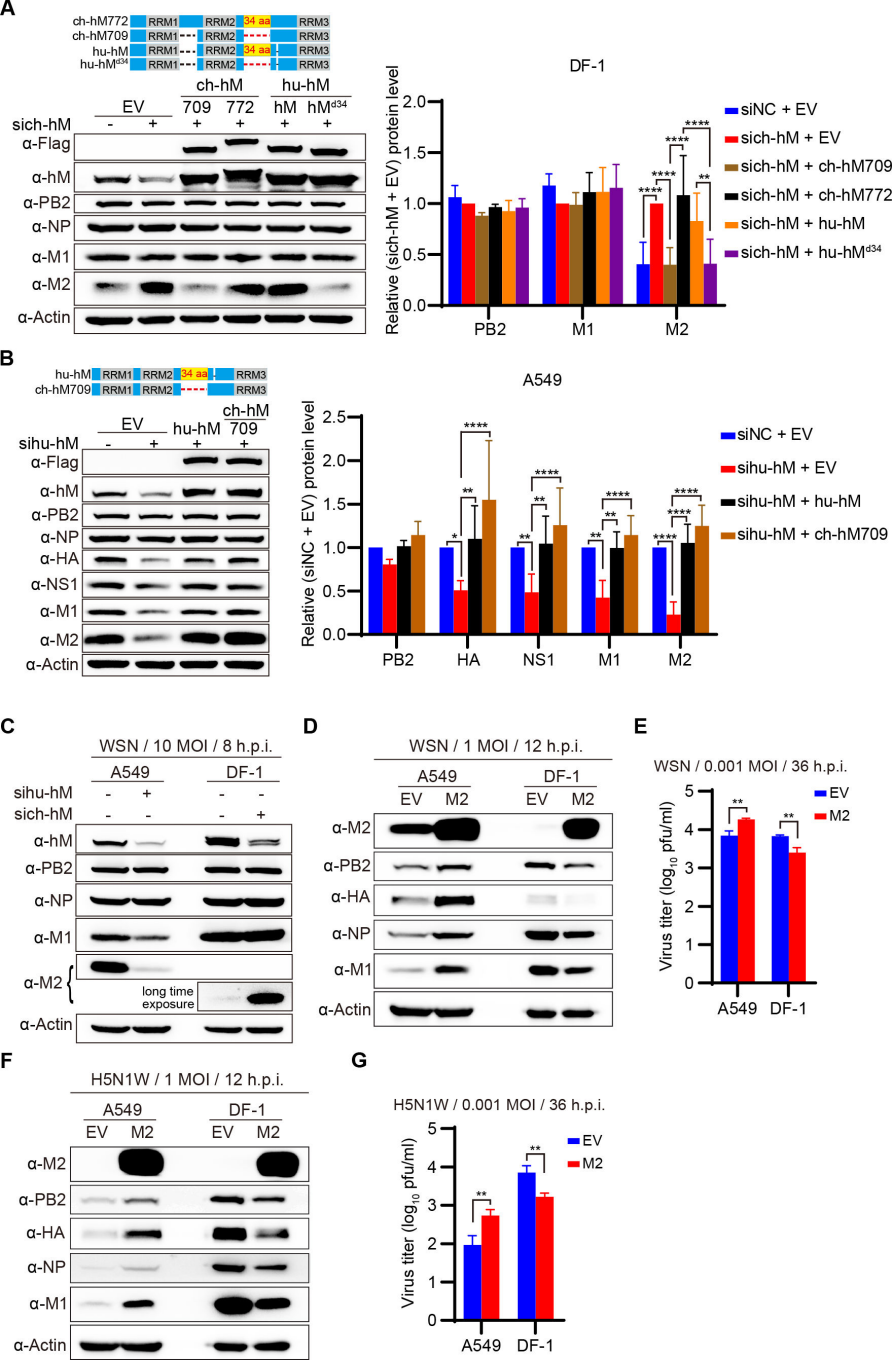
Species-specific regulation of M2 expression by hnRNPM influences IAV replication in different host cells. (**A**) Overexpression of chicken or human hnRNPM differentially regulates M2 protein expression in sich-hnRNPM-treated DF-1 cells. DF-1 cells treated with either sich-hnRNPM or siNC were transfected with the indicated plasmids. At 24 h post-transfection, the cells were infected with WSN virus at an MOI of 10. Cells were harvested 8 h post-infection and analyzed by western blotting. Quantification of viral proteins is shown in the right panel. **P < 0.01, ***P < 0.001, ****P < 0.0001. (**B**) Overexpression of human or chicken hnRNPM restores the differential expression of WSN virus protein in sihu-hnRNPM-treated A549 cells. A549 cells treated with either sihu-hnRNPM or siNC were transfected with the indicated plasmids. At 36 h post-transfection, the cells were infected with WSN virus at an MOI of 10. Cells were harvested at 7 h post-infection and analyzed by western blotting. Quantification of viral proteins is shown in the right panel. *P < 0.05, **P < 0.01, ***P < 0.001, ****P < 0.0001; ns, not significant. (**C**) Knockdown of host-specific hnRNPM differentially regulates M segment expression in human and avian cells. A549 and DF-1 cells were treated with control or targeting host-specific hnRNPM siRNAs and then infected with WSN virus for 8 h. Viral proteins were analyzed by immunoblotting. (D–G) Overexpression of WSN M2 differentially regulates IAV replication in human and avian cells. A549 and DF-1 cells were transfected with WSN M2 for 24 h and then infected with WSN or H5N1W at 1 MOI for 12 h (**D, F**) or at 0.001 MOI for 36 h (**E, G**). Virus titers in the supernatants were determined by plaque assays (**E, G**), and whole-cell lysates were analyzed by western blotting (**D, F**). *P < 0.05, **P < 0.01. Data are representative of at least three independent experiments. Means ± SD are shown in (**A, B, E, G**) (*n* ≥ 3).

The ch-hM738 and ch-hM709 isoforms, which are 34 amino acids shorter than the predicted ch-hM772 isoform, effectively inhibit M2 protein expression (Fig. 6M). Notably, hu-hnRNPM possesses exactly these additional 34 amino acids compared with the ch-hM738 and ch-hM709 (Fig. 6A). We then investigated whether these 34 divergent amino acids are responsible for the distinct regulatory mechanisms of hnRNPM in avian and human cells. We found that hu-hnRNPM did not inhibit M2 expression in DF-1 cells with ch-hnRNPM knockdown, whereas a hu-hnRNPM variant lacking these 34 species-specific amino acids suppressed M2 protein expression (Fig. 7A). This suggests that these 34 amino acids are critical for the species-specific regulation of M2 expression by hnRNPM in avian cells. In contrast, in A549 cells with hu-hnRNPM knockdown, both human and chicken hnRNPM restored the expression of HA, NS1, M1, and M2 proteins (Fig. 7B), indicating that hnRNPM does not exhibit species-specific regulation of IAV replication in human cells.

**Fig 8:**
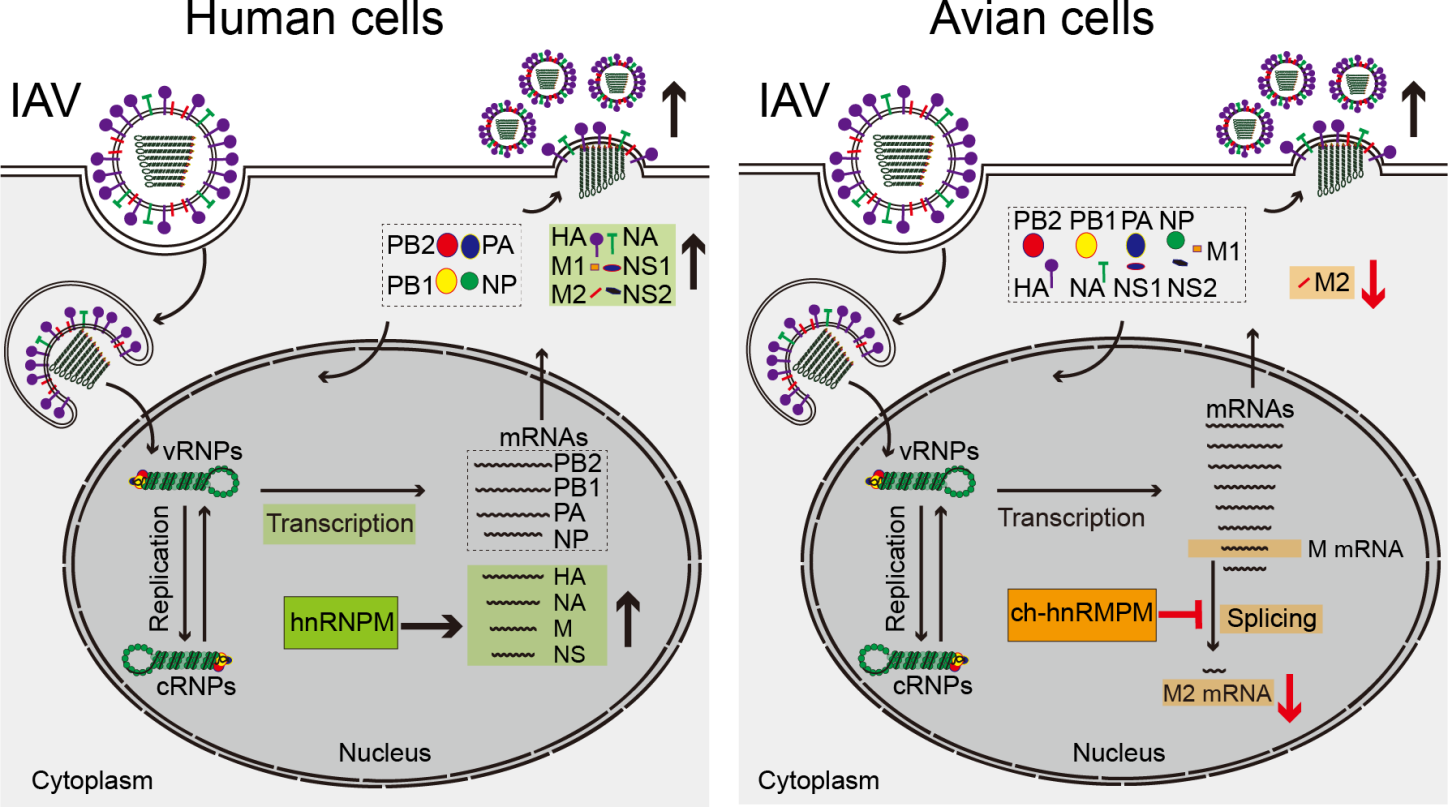
Proposed model for species-specific hnRNPM regulates influenza A virus replication in human and avian cells**.** During IAV infection, viral vRNPs are transported to the nucleus, where they drive viral RNA transcription and replication. Concurrently, the M and NS mRNAs undergo critical splicing events to generate M2 and NS2 mRNA, respectively. In human cells, hnRNPM regulates the transcription of the HA, NA, M, and NS segments, thereby enhancing their protein expression and promoting IAV virion production. In contrast, in avian cells, ch-hnRNPM specifically inhibits M segment mRNA splicing, thereby reducing M2 mRNA and protein levels, which in turn promotes IAV replication.

To further explore the role of hnRNPM in regulating IAV replication across different species, we simultaneously infected A549 and DF-1 cells with WSN virus. Knockdown of hu-hnRNPM in A549 cells resulted in reduced expression of both M1 and M2 proteins, whereas knockdown of ch-hnRNPM in DF-1 cells led to a dramatic increase in M2 protein without affecting M1 expression (Fig. 7C). Interestingly, the level of M2 protein was significantly lower in DF-1 cells than in A549 cells, suggesting that the required levels of M2 protein for IAV replication differ between human and avian cells. Excessive M2 production may enhance IAV replication in human cells but may be detrimental in avian cells. To test this hypothesis, we assessed the impact of overexpressing the WSN M2 protein in A549 and DF-1 cells on viral replication. Overexpression of M2 in A549 cells resulted in higher levels of intracellular viral proteins and increased WSN virus yield (Fig. 7D and E). In contrast, overexpression of M2 in DF-1 cells inhibited viral replication, confirming that excessive M2 production is detrimental to viral replication in avian cells. Additionally, the differential effects of M2 on IAV replication in A549 and DF-1 cells were further confirmed using the H5N1W virus (Fig. 7F and G). These findings suggest that the levels of IAV M2 protein are tightly regulated through distinct mechanisms in human and avian cells. In human cells, IAV utilizes hnRNPM to enhance the transcription of the M segment, thereby increasing the expression of both M1 and M2 proteins. In contrast, IAV relies on ch-hnRNPM to inhibit the splicing of excess M2 mRNA in avian cells, maintaining a low level of M2 protein that is optimal for viral replication (Fig. 8).

## DISCUSSION

The influenza A virus genome consists of multiple single-stranded, negative-sense RNA segments, enabling rapid adaptation across species. Transcription and splicing of the IAV genome in the nucleus of infected cells are tightly regulated to differentially produce optimal viral proteins for efficient progeny virion production. In this study, we revealed species-specific mechanisms of IAV transcription and splicing mediated by hnRNPM in human and avian cells. In human cells, hu-hnRNPM enhances IAV replication by promoting transcription of specific viral genomic segments (HA, NA, M, and NS). In avian cells, ch-hnRNPM sustains optimal M2 protein expression by suppressing excessive splicing of M2 mRNA, thereby facilitating IAV infection. These divergent regulatory effects directly influence IAV replication capacity in different host cells, highlighting a species-specific hnRNPM-mediated mechanism underlying viral host adaptation.

Host-specific proteins that support IAV replication are well-documented barriers to IAV cross-species transmission (27–29). For instance, human ANP32A, which lacks 33 amino acids compared with its chicken ortholog, fails to support the polymerase activity of avian IAV, thereby restricting its replication in human cells (30). In contrast, chicken ANP32A supports the polymerase activity of both human and avian IAV. Additionally, human BTN3A3 and MX1 regulate NP functions to inhibit avian IAV replication in human cells, whereas their chicken orthologs do not (28, 29). Human TRA2A, but not chicken TRA2A, restricts avian IAV replication but promotes human IAV replication in human cells by regulating the splicing of the M and NS segments (42). Our study extends this paradigm by revealing that human and chicken hnRNPM orthologs, differing by 34 amino acids, exert differential regulatory roles in IAV replication across species. Rather than overcoming the divergence in these orthologs, IAV adapts to their host-specific regulatory mechanisms. This suggests that cross-species adaptation requires the virus to evolve strategies that actively co-opt host-specific networks.

IAV replication in mammalian cell lines is characterized by differential viral protein expression, with higher levels of NP, HA, NA, M1, and NS1 and lower levels of polymerase proteins (PB2, PB1, and PA) (9–12). This pattern correlates with their roles in the virus life cycle and their relative abundances in virion formation (12). Previous studies have detailed host factors and viral proteins in the differential regulation of IAV protein production in M and MS mRNA splicing, viral mRNA nuclear export, and translation (15–19). However, host factors involved in regulating the differential protein expression at the transcriptional level have been rarely reported. Our findings show that hu-hnRNPM specifically regulates the transcription of HA, NA, M, and NS segments, providing evidence that IAV utilizes host factors to control differential transcription. This observation aligns with the viral protein synthesis dynamics in which PB2, PB1, PA, and NP segments are expressed early while HA, NA, M1, and M2 are produced relatively late in infection (6, 10, 43), highlighting that IAV utilizes hnRNPM to differentially regulate late gene transcription, thereby promoting efficient virus multiplication in human cells.

Interestingly, knockdown of human hnRNPM specifically reduces mRNA levels of the HA, NA, M, and NS segments, while increasing their corresponding vRNA levels in human cells (Fig. 4D and E). This observation suggests that hu-hnRNPM depletion selectively impairs vRNP-mediated transcription of these segments without affecting vRNP replication. The reduction in transcription may promote a shift of vRNPs from a transcription-competent to a replication-competent state (44, 45). In parallel, our group has recently shown that the sequentially expressed NS1 and NS2 proteins play key roles in regulating the transcription-to-replication switch during the viral life cycle (46). Therefore, reduced NS segment mRNA upon hu-hnRNPM knockdown likely disrupts the balance between transcription and replication. Notably, the increased mRNA levels of the PB2 segment observed in hu-hnRNPM knockdown cells at later stages of a single-cycle infection may result from the slow accumulation of NS1/NS2 proteins (Fig. 4B). Together, these findings highlight hu-hnRNPM as a critical host factor that modulates the dynamics of IAV transcription and replication in a segment-specific and temporally coordinated manner in human cells.

The relationship between the genetic sequences of influenza viruses and their replication efficiency has been extensively studied, with RNA sequences playing critical roles in various stages of the replication process (36–41). Each influenza RNA segment contains a highly conserved promoter region at both ends, followed by segment-specific NCRs and ORF (47). The packaging signals, formed by the NCRs and terminal ORF sequences, are crucial for the selective packaging of vRNPs into progeny virions (36, 48). Although NCRs regulate viral RNA transcription, replication, and segment-specific translation (38, 40, 41, 49–52), the role of ORF sequences in segment-specific transcription remains incompletely understood. Here, we provide evidence that ORF sequences of IAV play a critical role in segment-specific transcription, regulated by hu-hnRNPM in human cells. However, the precise mechanism by which hu-hnRNPM governs segment-specific transcription in an ORF sequence-specific manner remains unclear. Previous studies have suggested that within influenza vRNPs, NP-free regions with highly flexible secondary and/or tertiary structures are formed on the vRNP surface (53–56). These structural features may contribute to the differential regulation by hu-hnRNPM. Further studies are needed to elucidate the specific RNA features recognized by hu-hnRNPM.

The differential splicing of the M segment of IAV in human and avian cells has been proposed to play a key role in the host range determination (42, 57, 58). hnRNPM has been shown to directly bind to IAV M1 mRNA without regulating its splicing in human cells (15). We also found that hu-hnRNPM does not regulate M or NS segment splicing in human cells but promotes their transcription (Fig. 6L). In contrast, ch-hnRNPM inhibits M2 mRNA splicing in avian cells without affecting transcription (Fig. 6K). These observations further confirm that M segment splicing mechanisms differ between human and avian cells. Host factors, such as SRSF1, hnRNPK, NS1-BP, and SRSF5, have been reported to regulate M segment splicing in human cells (15, 59–61). However, host factors involved in regulating M segment splicing in avian cells remain unclear. Our data suggest that ch-hnRNPM, but not hu-hnRNPM, inhibits M2 mRNA splicing in avian cells, highlighting the role of host-specific hnRNPM in IAV cross-species adaptation.

Calderon et al. previously reported that avian-derived M segments, when compared with those from human-derived IAV strains, restricted viral growth and transmission in mammalian cells (57). This was linked to increased M2 expression and reduced M1 expression, with M2 overexpression causing intracellular autophagosome accumulation. Similarly, Bogdanow et al. observed striking differences in M1 and M2 expression levels between avian- and human-adapted IAV viruses in human cells (58). In contrast, our findings demonstrated that the same M segment from a single virus produces significantly different M2 protein levels in avian versus human cells (Fig. 7C). Literature data further support this, showing consistently higher M2 expression in human cells compared with avian DF-1 cells, irrespective of the virus subtype (62). Overexpressing M2 without altering splicing efficiency revealed that elevated M2 levels enhance IAV replication in human cells but inhibit replication in avian cells (Fig. 7D through G). These findings indicate that the optimal M2 protein levels for IAV replication differ between human and avian cells. In humans, M2 has been reported to play crucial roles in viral uncoating, vRNP packaging, and particle scission (63–65). Additionally, M2 facilitates virus budding by preventing lysosome degradation through the inhibition of autophagosome-lysosome fusion, thereby promoting virus protein transport to the membrane (66–69). A single nucleotide mutation altering M2 splicing efficiency in human cells increases M2 expression, enhancing A/duck/Sheyang/1/2005 (H5N1) virus replication, whereas reduced M2 levels diminish the infectivity of the WSN and PR8 viruses (42, 59, 62). In avian cells, however, the functional roles of M2 and the necessity for low M2 expression remain poorly understood. Our study confirms that excessive M2 protein is detrimental to IAV replication in avian cells, highlighting the distinct requirements for M2 regulation across host species. Nevertheless, the mechanisms by which variations in M2 protein expression impact viral replication remain unknown and warrant further investigation.

As the ongoing spread of highly pathogenic H5N1 avian influenza (HPAI H5N1) increases, concerns about potential cattle-to-human transmission grow (70–72). It has been reported that knockdown of hnRNPM inhibits the replication of the A/VN/1203/04 (H5N1) HALo virus and that increasing the expression of M2 relative to M1 enhances the replication of the A/duck/Sheyang/1/2005 (H5N1) virus in human cells (26, 42). Given the 99.3% similarity between cattle and human hnRNPM proteins, together with our findings observed in this study (Fig. 7F and G), hnRNPM likely contributes to high M2 expression, which is required for efficient replication of bovine H5N1 in both cow and human cells. Our study provides an early warning in this context.

In conclusion, our findings highlight distinct replication strategies employed by IAV across different host species and emphasize the mechanistic complexities underlying cross-species adaptation, particularly in balancing segment-specific transcription and splicing regulation. This study provides a foundation for further research into the molecular mechanisms that govern IAV cross-species adaptation.

## MATERIALS AND METHODS

### Viruses and cells

Influenza A/WSN/33 (WSN, H1N1) and A/Puerto Rico/8/34 (PR8, H1N1) viruses were kindly provided by Dr. Ervin Fodor (University of Oxford, UK). A/VN/1203/04 (H5N1) HALo virus was kindly provided by Dr. Adolfo García-Sastre (Icahn School of Medicine at Mount Sinai, USA). A/Quail/Hong Kong/G1/1997 (H9N2) virus was kindly provided by Dr. Chunfeng Li (Institute of Biophysics of the Chinese Academy of Sciences, China).

Human embryonic kidney cells 293T (HEK293T), human lung carcinoma cell line A549, Madin-Darby canine kidney (MDCK) cells, and chicken fibroblast cell line DF-1 were purchased from the American Type Culture Collection (ATCC, Manassas, VA). A549 *STAT1*-knockout cells were kindly provided by Dr. Jianwei Wang (Institute of Pathogen Biology of Chinese Academy of Medical Sciences, China). These cells were maintained in Dulbecco’s Modified Eagle Medium (DMEM) (Gibco), supplemented with penicillin (100 U/mL) (Gibco), streptomycin (100 µg/mL) (Gibco), and 10% fetal bovine serum (FBS) (Gibco) at 37°C in a humidified atmosphere containing 5% CO2.

### Antibodies

Immunoblot antibodies were diluted in 5% wt/vol milk-TBST according to the specified protocol. The antibodies used included: rabbit anti-influenza virus PB2 (produced in our laboratory), rabbit anti-influenza virus PB1 (GTX637313; Genetex), rabbit anti-influenza virus PA (GTX125932; Genetex), rabbit anti-influenza virus HA1 (11692-T62; Sino Biological), mouse anti-influenza virus NP (MAB8251; Sigma-Aldrich), rabbit anti-influenza virus M1 (GTX636677; Genetex), mouse anti-influenza virus M2 (sc-32238; Santa Cruz), rabbit polyclonal anti-influenza virus NS1 (GTX125990; GeneTex), mouse anti-hnRNPM (TA301557; Origene), mouse anti-DYKDDDDK (Flag) tag (RA1003-01; Vazyme), mouse anti-β-actin (HC201; TransGen), rabbit anti-tandem affinity purification (TAP) tag (sc-25768; Santa Cruz), rabbit anti-human STAT1 (p84/p91) (sc-346; Santa Cruz), rabbit anti-IRF3 (11904; CST), rabbit anti-phosphorylated IRF3 (S386) (ab76493; Abcam), goat anti-mouse IgG (AS003; Abclonal), and goat anti-rabbit IgG (AS014; Abclonal).

### Plasmids

The pHW2000 eight-plasmid rescue system for influenza virus A/WSN/1933 (WSN, H1N1) has been previously described (73). The HA vRNP reconstitution system for WSN virus (pcDNA-PB2, pcDNA-PB1, pcDNA-PA, pcDNA-NP, and pPOLI-HA) was kindly provided by Dr. Ervin Fodor (Oxford University, Oxford, UK) (74). The plasmid containing the synonymous mutant of the PR8 M1 gene was kindly provided by Dr. Qi Jianxun (Institute of Microbiology, Chinese Academy of Sciences, China). Plasmids pcDNA-PB2-TAP, pPOLI-M, pPOLI-PB2, pPOLI-PB2(GFP), pPOLI-NA(GFP), pPOLI-PB2(M), pPOLI-PB2(M1), pPOLI-PB2(NA-Flag), pPOLI-PB2(M1^mut^-Flag), pCAGGS-WSN M2, pCAGGS-human-hnRNPM, pCAGGS-chicken-hnRNPM, and the specific synonymous mutants were constructed using standard molecular biology techniques.

### Tandem affinity purification and LC-MS/MS analysis

293T cells were transfected with specific expression plasmids (pcDNA) encoding polymerase subunits (PA, PB1, and either PB2 or PB2-TAP fusion protein), NP, and a plasmid transcribing HA vRNA (pPOLI-HA) to express HA vRNP. Cells were harvested 24 h post-transfection and lysed with lysis buffer (50 mM Tris-HCl [pH 8.0], 200 mM NaCl, 0.5% NP-40, 25% glycerol, 1 mM β-mercaptoethanol, 1 mM phenylmethylsulfonyl fluoride [PMSF], and 1% protease inhibitors). The cell lysates were centrifuged, and the supernatants were incubated with the specified TAP antibody overnight at 4°C, followed by incubation with IgG Sepharose (17–0969-01; Axygen) for 2 h at 4°C. The beads were washed with binding buffer (50 mM Tris-HCl [pH 8.0], 150 mM NaCl, 0.2% NP-40, 1% glycerol, 1 mM PMSF). Bound proteins were then released by cleavage using tobacco etch virus (TEV) protease (12575023; Invitrogen). Purified proteins were separated by 8% SDS-PAGE and visualized by silver staining. TAP-purified samples were identified by liquid chromatography-mass spectrometry (LC-MS) as previously described (75).

### Plaque assay

MDCK cells (3 × 10^5^) were seeded into 12-well plates and incubated at 37°C with 5% CO_2_ for 18 h. Each virus was serially diluted from 10^−1^ to 10^−7^ in DMEM media containing 0.5 µg/mL TPCK-trypsin and 1% bovine serum albumin (BSA) (V900933; Sigma). After washing the cells with PBS, they were infected with viruses and incubated at room temperature for 1 h. The unbound viruses were removed with PBS, and the cells were covered with a media mixture of 1% low-melting agarose (A9045; Sigma) containing 0.5 µg/mL TPCK-trypsin and 0.5% FBS. The plates were then inverted and cultured at 37°C with 5% CO2 for 3 days. The cells were then fixed with 4% formaldehyde (P1110; Solarbio) overnight at 4°C, stained with 1% crystal violet (C8470; Solarbio) for 5 min at room temperature, and washed carefully with water. The number of plaque-forming units (PFU) was counted, and the virus titers were calculated based on the dilution factors.

### RNA interference and DNA transfection

Cells were transfected with siRNAs at 3 nM or 6 nM using Lipofectamine RNAiMAX (13778150; Invitrogen) according to the manufacturer’s protocol. Knockdown efficiency assays and viral infection studies were performed after transfection for 36 h or 48 h. Knockdown efficiency was assessed by RT-qPCR and western blot analysis. Specific siRNAs used included:

Human sihnRNPM-1# 5′-GCUGCUAUUUGAUAGACCA-3′;

Human sihnRNPM-2# 5′-CCAACAAUCUGGAGCGGAU-3′;

Chicken sihnRNPM-1# 5′-GCACUGUCUUUGUUGCAAA-3′;

Chicken sihnRNPM-2# 5′-GGAAAUCGCUUUGAGCCAU-3′; and

Negative control siRNA 5′-UUCUCCGAACGUGUCACGU-3′.

Cells were transfected with siRNA for 6 h, followed by plasmid DNA transfection using Lipo8000 (C0533; Beyotime) according to the manufacturer’s instructions. Viral infection studies were performed 36 h after the initial siRNA transfection.

### Western blot

Cells were lysed on ice for 30 min using RIPA lysis buffer (P0013B; Beyotime) supplemented with 1% protease inhibitor cocktail (11836170001; Roche). The cell lysate was collected and centrifuged at 12,000 rpm for 15 min to remove cellular debris. The supernatants were then separated by SDS-PAGE on an 8%–15% gradient gel and transferred onto nitrocellulose membranes (66485; PALL). After blocking to prevent nonspecific binding, the membranes were incubated with specific antibodies. Protein bands were visualized using an enhanced chemiluminescence detection kit (P10200; NCM Biotech) and imaged with a ChemiScope 6100 imaging system (CLINX; China). Quantification of protein bands was performed using ImageJ software.

### RT-qPCR

Total RNA was extracted using TRIzol reagent (15596–018CN; Thermo Fisher) according to the manufacturer’s instructions. cDNA was synthesized using the Reverse Transcription cDNA Synthesis Kit (TSK302M; TSINGKE) with oligo(dT) or specific primers, and then diluted 6-fold to serve as a template for qPCR. Quantitative PCR (qPCR) was conducted in technical duplicate or triplicate using gene-specific primers and SYBR Green premix (A25742; Thermo Fisher). Real-time data were collected using QuanStudio 7 Design and Analysis software (4485701; Thermo Fisher), normalized against internal standards using the 2^^(-ΔΔCt)^ method, and analyzed using GraphPad Prism 8 software.

### Primer extension analysis

Influenza virus RNAs (vRNA and mRNA/cRNA) were simultaneously detected using primer extension analysis as previously described (76, 77). Total RNA was extracted using TRIzol reagent, and cDNA was synthesized using the SuperScript III Reverse Transcriptase Kit (18080044; Invitrogen) with specific primers labeled with ^32^P. Transcription products were separated on 6% polyacrylamide gels containing 7 M urea, visualized by autoradiography, quantified with ImageJ, and analyzed using GraphPad Prism 8 software.

### Co-immunoprecipitation (CO-IP)

A549 cells were infected with WSN virus (MOI = 1) for 12 h post-infection. The cells were then lysed on ice for 30 min using lysis buffer (50 mM HEPES [pH 7.2–7.4], 150 mM KCl, 2 mM EDTA, 1% NP40, 0.5 mM DTT, 1 mM NaF, 1 mM PMSF, and 1% protease inhibitors). Lysates were collected and centrifuged at 12,000 rpm for 15 min to remove cell debris. Prepared Dynabeads Protein G (10006D; Invitrogen) were incubated with the indicated antibody for 30 min at 4°C, followed by incubation with the cell lysates for 1 h at 4°C. The Dynabeads were washed three times with washing buffer (50 mM Tris-HCl [pH 7.4], 150 mM NaCl, 0.5% NP40, 1 mM MgCl_2_, 1 mM PMSF). Finally, the bound proteins were eluted with an Elution Buffer (10006D; Invitrogen) and analyzed by western blotting.

### Statistical analysis

All statistical analyses were performed using Prism software (GraphPad). Data are presented as mean ± standard error of the mean (SEM) in bar graphs. One-way or two-way ANOVA with Dunnett’s correction was used for multiple comparisons. Student’s *t*-test was used for two-group comparisons. Statistical significance is indicated as follows: *P < 0.05, **P < 0.01, ***P < 0.001, ****P < 0.0001.

## Data Availability

All data from this study are included in the paper and are available from the corresponding author upon reasonable request.
